# Human complex mixture analysis by “FD Multi-SNP Mixture Kit”

**DOI:** 10.3389/fgene.2024.1432378

**Published:** 2024-09-27

**Authors:** Anqi Chen, Lun Li, Junfei Zhou, Tiantian Li, Chunyan Yuan, Hai Peng, Chengtao Li, Suhua Zhang

**Affiliations:** ^1^ Institute of Forensic Science, Fudan University, Shanghai, China; ^2^ Institute for Systems Biology, Jianghan University, Wuhan, Hubei, China; ^3^ School of Life Sciences, Jianghan University, Wuhan, Hubei, China; ^4^ Shanghai Key Laboratory of Forensic Medicine, Shanghai Forensic Service Platform, Ministry of Justice, Academy of Forensic Science, Shanghai, China

**Keywords:** genetic markers, multi-SNPs, individual identification, mixture detection, forensic

## Abstract

**Introduction:**

Multiple linked single nucleotide polymorphisms (SNPs) have shown potential in personal identification and mixture detection. However, the limited number of marker and sequencing errors have obstructed accurate DNA typing.

**Methods:**

To develop more candidate loci, the diversity value (D-value) was introduced as a new parameter for screening the novel polymorphic multiple linked-SNP markers, referred to as multi-SNP. In this study, a “FD Multi-SNP Mixture Kit” comprising 567 multi-SNPs was developed for mixture detection. Additionally, a new computational error correction method was applied as a quality control approach for sequencing data.

**Results:**

The results demonstrated higher typing success rates than the conventional CE typing method. For single-source DNA, approximately 70–80 loci were detected with a DNA input of 0.009765625 ng. More than 65% of the minor alleles were distinguishable at 1 ng DNA with a frequency of 0.5% in 2- to 4-person mixtures.

**Conclusion:**

This study offers a polymorphic and high-resolution detection method for DNA genotyping and complex mixture detection, providing an alternative strategy for addressing challenging mixed DNA traces.

## 1 Introduction

Mixture identification is a demanding task in the field of DNA analysis. The detection and analysis of complex mixtures have been among the most challenging problems faced by forensic DNA laboratories (Butler, 2023). Over the past decade, researchers have conducted studies on the collection, detection, and analysis of mixed samples, achieving some success in identifying two-person mixed samples. However, the examination and analysis of complex mixtures using existing techniques remain unsatisfactory in terms of their efficiency.

The conventional capillary electrophoresis-based short tandem repeat (CE-STR) typing method is the most commonly used approach for DNA identification (Chen et al., 2021a). However, STR profiles of mixed samples often show the coexistence and superposition of alleles from each contributor (Chen et al., 2021b; Chen et al., 2020). The generation of stutter, imbalance of alleles, random amplification effects, and interpretation of background signals all affect the possible typing combinations, making it more challenging for forensic scientists (Wu et al., 2019; Chen et al., 2022). Studies have shown that the STR profile might be incomplete if the minor contributor’s DNA proportion is less than 5%–20% (Chen et al., 2022; Zheng et al., 2019; Chen et al., 2019), which has limited its application in the identification of low template DNA. The International Society of Forensic Genetics (ISFG) also states that detection using autosomal STR locus is not completely reliable when the proportion of the minor contributor in a mixed two-person sample is less than 5% (Crespillo et al., 2014).

Compared to STR, single nucleotide polymorphism (SNP) is an attractive genetic marker because of its simplistic and easily readable typing results, and the lack of stutter peaks in the typing process (Babol-Pokora and Berent, 2008; Freire-Aradas et al., 2012; Brown et al., 2017). Although the SNP loci have limitations in their discriminatory power, several studies revealed that SNPs have some usefulness in the analysis of mixed samples (Isaacson et al., 2015; Kaur et al., 2014). Kidd et al. (Kidd et al., 2017; Kidd and Speed, 2015) first introduced the concept of microhaplotype loci (microhaps, MHs), which contain two or more closely linked SNPs. MHs provide significantly more information than single SNP markers (Aly and Sabri, 2015). They have demonstrated their utility in various fields, such as identifying missing persons, determining paternity, and detecting mixed samples. The innovations in haplotype phasing methods are essential to the effectiveness of MH analysis. Statistical phasing allows for the inference of haplotypes from population data (Kidd et al., 2022; Wu et al., 2021), family-based phasing leverages known familial relationships to improve accuracy (Wei et al., 2024), and long-read physical phasing directly sequences longer DNA fragments, preserving the linkage between variants (Kureshi et al., 2020). These advances collectively enhance the resolution and reliability of MH analysis, making it a powerful tool for complex genetic investigations. To date, an increasing number of MHs have been identified, but distinguishing minor alleles with frequencies below 1.5% remains challenging (Tao et al., 2022). Furthermore, the error rates of Illumina sequencing machines are approximately 0.1% per bases sequenced (Stoler and Nekrutenko, 2021), which suggests the low-abundant signals are likely to be masked. To further address the challenges mentioned above, it is necessary to develop methods for marker screening as well as accurate allele calling.

In this study, we developed a new solution named “FD Multi-SNP Mixture Kit”, which screened a specific category of SNP-based genetic markers and applied a novel strategy to call SNP alleles. Unlike the classic MHs selection approach (Kidd et al., 2017; Kidd and Speed, 2015), the new markers (referred to as multi-SNP) include combinations of two or more SNP loci that possess the D (diversity) value within 75 base pairs (bp). The good polymorphism and short amplification fragments might make them suitable for forensic samples. In addition to marker screening, new strategies for analyzing multi-SNP marker data were employed to decode the identity information of minor contributors. The results demonstrated that the approach was highly sensitive and effective for analyzing mixed samples.

## 2 Materials and methods

### 2.1 Genome-wide screening of multi-SNPs

The multi-SNPs markers were selected in accordance with our previous research with a few minor modifications (Fang. et al., 2021). The SNPs were obtained from the publicly accessible whole-genome sequence data of 91 Chinese Han individuals from the 1,000 Genomes Project (BioProject accession: PRJEB11005). A window size of 75 bp was chosen to accommodate complex DNA characteristics, such as trace amounts or high degradation. To select highly discriminatory multi-SNPs, the polymorphism within the window was evaluated using the D-value, which was calculated by t⁄((N¦2), where (N¦2) represented the total number of pairwise combinations that could be made within a population of N individuals, and t denoted the number of distinguishable pairings based on at least two single nucleotide variations found within the window. The windows susceptible to amplification, sequencing, and computational analysis errors, such as sequences comprising PolyN or tandem repeats, were filtered out. Only windows with a D-value of ≥ 0.6 were submitted to https://ampliseq.com for multiplex primer design. The amplicon length was set to be below 140 bp so that a single sequence read could cover the entire amplicon.

### 2.2 Library construction and sequencing

For each sample, the sequencing library was constructed using 5 μL of DNA following the protocol of the MGIEasy Universal DNA Library Prep Set (MGI-Tech, China), with the number of PCR cycles set to 28. Ten additional PCR cycles were then performed to anneal eight-nucleotide barcode sequences specific to each sample for multiplexing during sequencing. Finally, the pooled libraries were sequenced on the Illumina NovaSeq X platform to obtain 150 bp paired-end reads.

### 2.3 DNA samples

A total of 409 unrelated individuals from the Chinese Han population were recruited for the study. All samples were collected upon the approval of the Ethics Committee at the Academy of Forensic Science, Ministry of Justice, China. Each participant provided written informed consent to participate in the study. Genomic DNA (gDNA) samples were extracted from 200 μL of peripheral blood by using a QIAamp DNA Blood Kit (Qiagen, Netherlands). The gDNA was extracted according to the manufacturer’s instructions and quantified using a Qubit fluorometer (Life Technologies, United States). All extracted DNA was stored at −80 °C until use.

Two single-source DNA samples (one male and one female) with known genotypes were employed for the sensitivity study of the “FD Multi-SNP Mixture Kit”. The gDNA samples were diluted with molecular biology grade water (Phenix, United States). Ten different DNA inputs were prepared as follows: 5 ng, 2.5 ng; 1.25 ng, 0.625 ng, 0.3125 ng, 0.15625 ng, 0.078125 ng, 0.0390625 ng, 0.01953125 ng and 0.00976563 ng. In total, 60 samples (2 single-source DNA samples × 10 input amounts × 3 replicates) were sequenced in the sensitivity study.

In this study, two batches of artificial DNA mixtures were prepared. The first batch was prepared to compare the conventional CE-STR method and the next-generation sequencing (NGS) method of the “FD Multi-SNP Mixture Kit”. This batch included thirteen 2-person mixtures, eight 3-person mixtures, six 4-person mixtures and ten 5-person mixtures (Supplementary Table S3). The second batch was prepared to evaluate the mixture detection performance of the “FD Multi-SNP Mixture Kit”. It included seventy-five contrived mixture samples in total: twenty-five 2-person mixtures, twenty-five 3-person mixtures, twenty-three 4-person mixtures, one 5-person mixture and one 10-person mixture (Table 1). All participants were initially tested for all multi-SNPs using the “FD Multi-SNP Mixture Kit” and the allele typing results were obtained.

**TABLE 1:** Summary of the DNA mixtures prepared for the mixture study (n = 75).

Mixture	Ratios (%)	Total input amount (ng)						
2-person	90:10	5	2	1	0.5	0.2	0.1	0.05
	95:5	5	2	1	0.5	0.2	0.1	
	98:2	5	2	1	0.5	0.2		
	99:1	5	2	1	0.5			
	99.5:0.5	5	2	1				
3-person	70:20:10	5	2	1	0.5	0.2	0.1	0.05
	85:10:5	5	2	1	0.5	0.2	0.1	
	94:4:2	5	2	1	0.5	0.2		
	97:2:1	5	2	1	0.5			
	98.5:1:0.5	5	2	1				
4-person	50:20:20:10	5	2	1	0.5	0.2	0.1	0.05
	65:20:10:5	5	2	1	0.5	0.2	0.1	
	86:8:4:2	5	2	1	0.5	0.2		
	93:4:2:1	5	2	1				
	96.5: 0.2:0.1:0.05	5	2					
5-person	20:20:20:20:20	5						
10-person	10:10:10:10:10:10:10:10:10:10	10						

### 2.4 Bioinformatics

For each sample, the raw reads were mapped to the human reference genome using bowtie2 (Langmead and Salzberg, 2012) (version 2.1.0), and the unmapped or partially mapped reads were discarded in the further studies. For the fully mapped read, the nucleotide sequence spanning a multi-SNP locus was taken as its allele.

To lessen technical errors, several measures were employed as follows:

(1) Any variants located within or two base pairs from repeat sequences, consecutive mismatches, or indels were ignored to reduce the errors due to improper alignment. Since sequencing errors happen independently between reads, it’s unlikely that both reads of a pair have incorrect but identical alleles. Therefore, only the paired-end reads with identical alleles were retained to minimize false alleles brought about by sequencing errors.
(2) An iterative procedure was performed to identify true alleles from PCR artifacts: 1) All detected alleles in one multi-SNP locus were gathered, and the allele with the greatest read number was designated as the major allele. 2) For the remaining alleles (denoted as candidate alleles), they were temporary presumed as products of the major allele (e.g., PCR errors). The reads number of each candidate allele was modeled as a binomial distribution of total read counts of the major and candidate alleles and the error rate (e). If the counts number (c) of a candidate allele was excessive, resulting a significantly low P (X ≥ c), the null hypothesis that the allele was merely an artifact should be rejected. When more than one candidate alleles were tested, the multiple test correction was conducted, and the candidate alleles with false discovery rate (FDR) < 0.5% were deemed as the true minor alleles. 3) In the subsequent iterations, the authenticity of the remaining candidate alleles was assessed similarly to the first round, with the exception that for each candidate allele, the true allele that was the most similar was taken as the template of the erroneous amplification. The procedure terminated when no more minor alleles were found.

The PCR error rates may be affected by the nucleotide composition of locus, thus locus-specific error rates should be employed in the statistical models. Besides, since the errors happened independently across reads, the odds of multiple errors occurring within one read sequence was lower than that of a single one, making it necessary to use an error rate specific to on the number of single nucleotide variants (n) between the candidate allele and true allele. All alleles except the two alleles with most supporting reads at the multi-SNP loci of individual samples were produced by technical errors, and the variants on these alleles were regarded as false. In this study, for a multi-SNP locus l, an error profile was estimated from 409 Chinese Han individual samples, and the parameter el(n) was the average ratio of reads that allele bearing n false SNPs.

For each individual sample, loci with major allele read depths greater than 50 were further genotyped. The homozygous locus was recognized if the minor alleles with α values (the ratio of supporting read count of the minor allele to that of the major allele) less than 0.05, and the major allele was taken as the genotype. In contrary, if a locus had only one allele with an α value between 0.05 and 0.2, it was determined as heterozygous with the major allele and the minor allele as its genotype. Otherwise, the locus failed genotyping and was discarded from further studies.

### 2.5 Statistical analysis

The ACR value at heterozygous genotypes was calculated as the smaller number of depths at one allele compared to the other. Allele frequencies were calculated using the counting method. The other forensic statistical parameters, including expected heterozygosity (He), observed heterozygosity (Ho), match probability (MP), discrimination power (DP) and probability of exclusion (PE), were calculated using Power Stats v12 software (Promega, United States). Hardy–Weinberg Equilibrium (HWE) was estimated using Arlequin 3.533 software. Detection rate was applied to assess the concordance for both the single- and multi-sourced DNA samples, calculated as the ratio of the number of alleles matched to the total number of alleles detected.

### 2.6 Conventional CE-STR typing

The STR profiles of the first batch of artificial DNA mixtures were determined using a PowerPlex^®^21 kit (Promega, United States), which includes 20 autosomal STRs and one sex-linked polymorphism Amelogenin. Fluorescence multiplex PCR was performed according to the manufacturer’s instructions and the amplified products were separated on a 3100 ABI Prism Genetic Analyzer (Applied Biosystems, United States). Genotyping was per-formed using GeneMapper software (Applied Biosystems, United States), and all results were checked by an experienced technician.

## 3 Results and discussion

The MHs, which exhibit a high degree of polymorphism, have been shown to have significant potential in mixture deconvolutions (Chen et al., 2019). To identify more polymorphic multi-allelic markers, we proposed the D-value as a new parameter. Using this approach, a total of 519 multi-SNP markers were identified, including 157 two-linked SNPs, 246 three-linked SNPs, 91 four-linked SNPs and 25 five-linked SNPs. To further enhance polymorphic diversity, an additional 48 single SNP markers were also incorporated (Table 2). As a result, a toolkit named the “FD Multi-SNP Mixture Kit” was developed for high-throughput DNA profiling and mixture detection.

**TABLE 2:** Loci constitution of the “FD multi-SNP Mixture Kit”.

Category	Number	Percentage (%)
Single SNP	48	8.47
2-linked SNPs	157	27.69
3-linked SNPs	246	43.39
4-linked SNPs	91	16.05
5-linked SNPs	25	4.41

### 3.1 Sequencing performance of the “FD Multi-SNP mixture kit”

**FIGURE 1:**
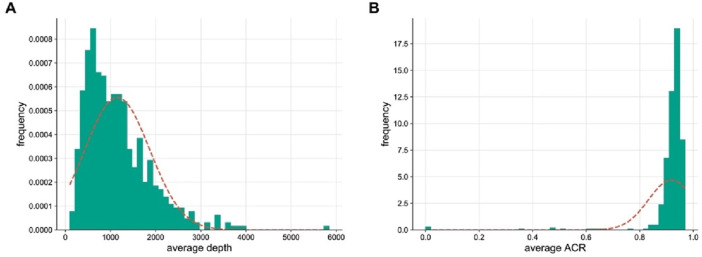
Sequencing depth and ACR of the “FD Multi-SNP Mixture Kit”. **(A)** Coverage depth. **(B)** Distribution of loci across different ACR ranges.

To evaluate the performance of the panel, we sequenced the DNA samples of 409 unrelated individuals from the Chinese Han population. For single-source sample, the panel achieved an allele coverage ratio of 98.21%, indicating that there were sufficient loci for forensic identification. The average sequencing depth was 1150.87 ± 716.64×, ranging from 99.19 ± 35.53× to 5841.08 ± 929.49×. The depth of CHM527 was the highest, followed by CHM277 (3917.78 ± 2041.55×), CHM511 (3856.50 ± 1857.29×), CHM528 (3712.10 ± 1723.12×) and CHM101 (3629.66 ± 1502.32×). The depths of CHM578, CHM044, CHM303, CHM416 and CHM085 were the lowest, with depths below 200×. Although sequencing depths varied across loci, the lowest depth remained at approximately 100× (Figure 1A). Jiang et al. (2019) argued that deeper sequencing depths lead to higher typing accuracy. In this study, the sequencing depths of the markers were comparable to those in published SNP-based sequencing studies (Guo et al., 2017; Tao et al., 2021), ensuring good data quality for further analysis.

The average ACR of the panel was 0.92 ± 0.05, which is excellent according to the current knowledge (Figure 1B). The forensic community defines a DNA mixture as a biological sample derived from two or more contributors (Bieber et al., 2016). Thus, a good intralocus balance is more favorable for mixture deconvolution (Pang et al., 2020). Due to the characteristics of multi-SNP markers, a cold case from several decades ago was resolved (Chen et al., 2024). The case involved a sample from a campstool, which was suspected to be the potential weapon in a homicide. DNA extraction and STR profiling of the campstool revealed that the DNA was a mixture. However, the results from CE-STR were fraught with uncertainty and controversy. In contrast, analysis using NGS with the “FD Multi-SNP Mixture Kit” indicated that the probability of the suspect’s DNA being present on the campstool was as high as 99.999159%. This result contradicted the suspect’s statement and was instrumental in solving the case, demonstrating the effectiveness of the FD Multi-SNP Mixture Kit for degraded and trace DNA mixtures in forensic investigations.

### 3.2 Marker detection performance of the “FD Multi-SNP mixture kit”

**FIGURE 2:**
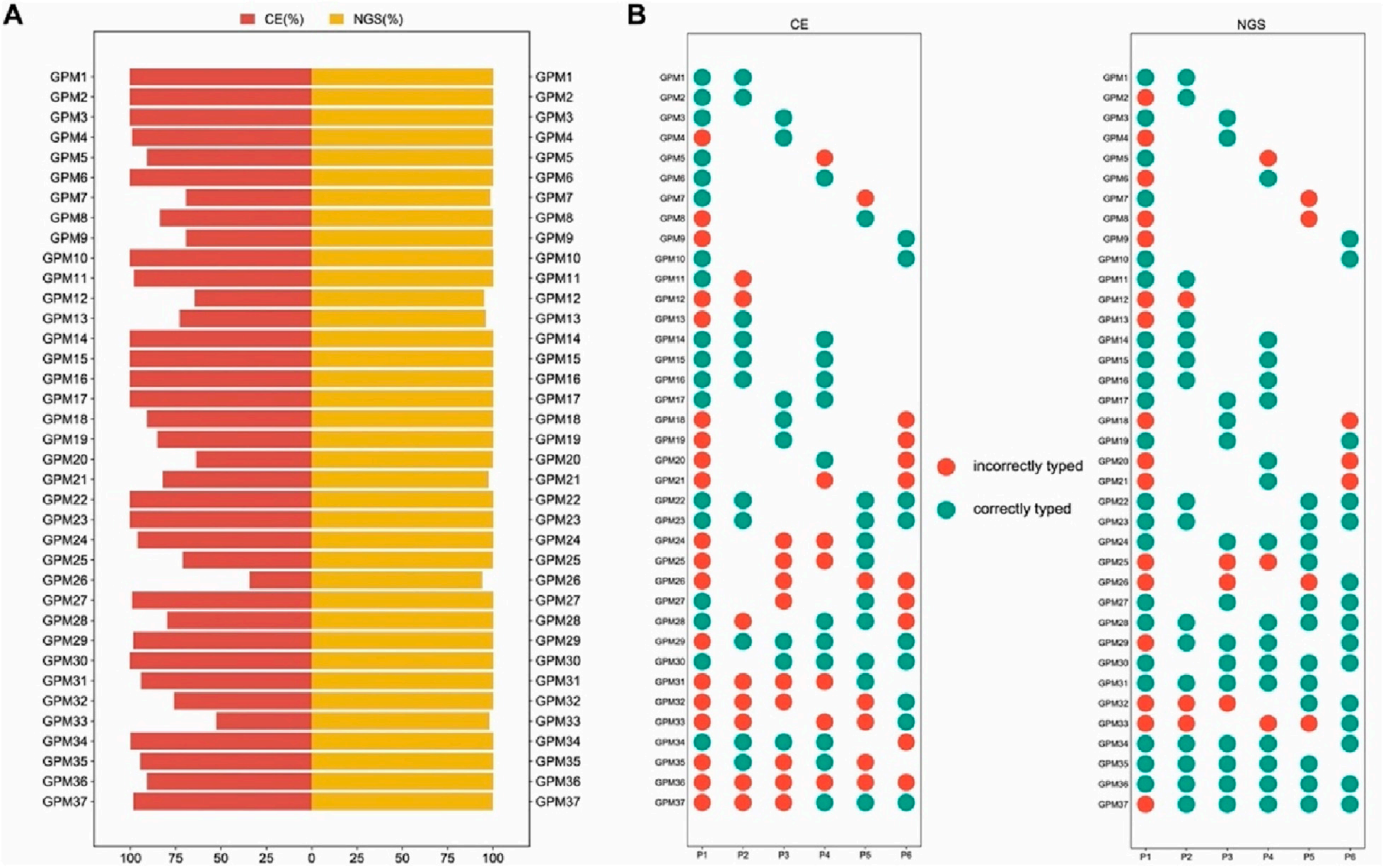
Comparison of detection rates between the two methods for 37 mixed samples. **(A)** Comparison of the overall detection rates of the CE typing methods. **(B)** Typing accuracy of each contributor in the mixture samples (P1, P2, P3, P4, P5 and P6 represent six unrelated individuals).

For approximately 30 years, STR markers with 3-4 bp repeats have been considered the conventional marker in forensic genetics. However, the stochastic effects of this method would likely to reduce the success rate of low-input DNA analysis (Nwawuba Stanley et al., 2020). To investigate whether multi-SNP markers are more suitable for mixture analysis, a back-to-back comparison was made between the standard CE-STR method and the NGS of “FD Multi-SNP Mixture Kit”. The result showed higher detection rates (93.76%–100%) with the NGS of “FD multi-SNP Mixture Kit”, while lower detection rates (33.93%–100%) were observed with the conventional approach (Figure 2A). The higher the resolution of detection, the more reliable the mixture deconvolution. In this study, the NGS method of “FD Multi-SNP Mixture Kit” accurately detected more alleles, suggesting that it may provide more opportunities for further interpretation of DNA mixture. In addition, the success rates of minor contributors were lower than those of major contributors, regardless of the typing method (Figure 2B, Additional file: Supplementary Table S3), further demonstrating that absolute amounts of DNA play a critical role in detection rates and sequencing performance of the “FD Multi-SNP Mixture Kit”.

### 3.3 Sensitivity performance of the “FD Multi-SNP mixture kit”

**FIGURE 3:**
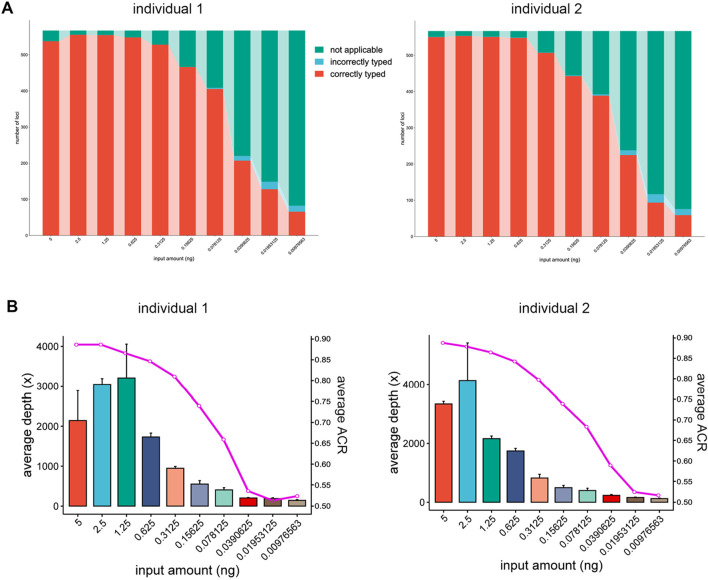
Sensitivity study of the “FD Multi-SNP Mixture Kit”. **(A)** Counts of loci detected at different input amounts. **(B)** Sequencing depths and average ACR for different DNA input amounts.

To further investigate the sensitivity of this panel, a series of diluted DNA samples were tested. The fluctuating trends were observed in the counts of loci detected from 5 ng, 2.5 ng, 1.25 ng and 0.625 ng of DNA input. Among these, approximately 95%–97% of the loci were correctly typed. The low standard deviations within the same DNA inputs showed comparable results in the triplicates. The number of loci detected decreased when the input amount was less than 0.625 ng. At 0.3125 ng DNA, more than 90% of the profiles could be typed correctly. However, many allelic dropouts began at 0.15625 ng DNA, and only 15% of the loci could be detected when the input was reduced to 0.00976563 ng. The abundance of loci in this panel provide more opportunities for DNA identification (Bruijns et al., 2018). For this panel, over 70 loci were detected with an input of 0.00976563 ng DNA (Figure 3A). It has been reported that 40 to 60 SNPs have a comparable power of 13–15 STRs (Butler et al., 2007), and the multi-SNP markers have more alleles di-allelic SNPs. Therefore, the remaining markers should have retained a comparable discrimination power of 20–23 STR loci.

In this study, sequencing depths of over 1,000× were measured when the input amount was above 0.3125 ng, and the ACR remained above 0.7 even when the DNA input was as low as 0.15625 ng. A dramatic decrease in depth and ACR occurred when the input was reduced to 0.0390625 ng. Under this circumstance, the average depth was no more than 300×, and the average ACR was only 0.5. To fully utilize the flow cells and ensure consistent sequencing depth for all samples, diluted libraries of variable concentration should be volumetrically pooled (Bruinsma et al., 2018). Based on the observed depth variations, the amount of 5ng to 0.625 ng of DNA could potentially be attributed to pipetting errors during library normalization. Compared to sequencing depth, the ACR values were less sensitive to the amount of DNA input. High quality ACR values (>0.7) could be maintained even when the input DNA was reduced to 0.15625 ng (Figure 3B). Balanced ACR is advantageous for distinguishing components from a DNA mixture (Pang et al., 2020), and the results demonstrated its potential applications in the detection and deconvolution of low-amount DNA.

### 3.4 Mixture detection performance of the “FD Multi-SNP mixture kit”

**FIGURE 4:**
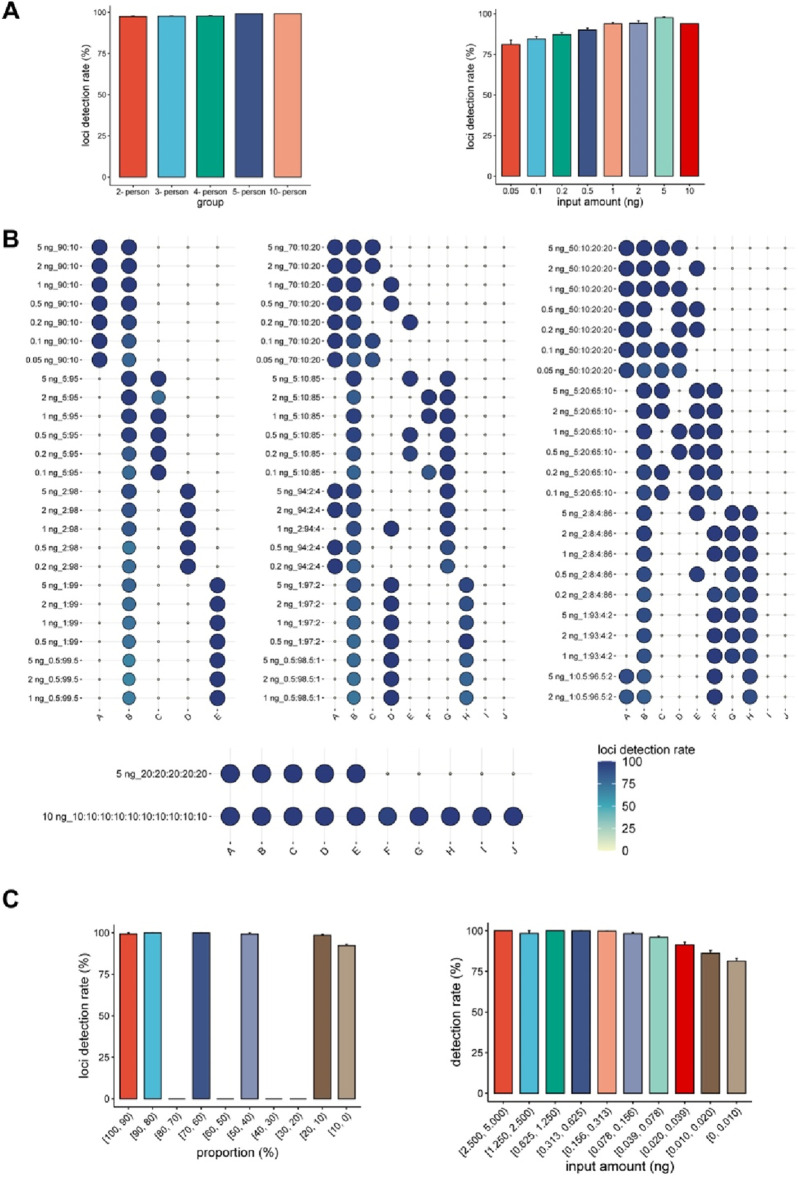
Detection performance of the “FD multi-SNP Mixture Kit”. **(A)** Histogram of the detection rates in the mixtures with different numbers of contributors and different DNA inputs. The *x*-axis represents the number of individuals (left) and the total input amounts in the mixtures, while the *y*-axis shows the average detection rates of the samples. **(B)** Heatmap showing the detection rate for each of the DNA mixtures in the study (N = 75). **(C)** Detection rate of loci from contributors at different proportions and different input amounts. The *x*-axis represents the DNA amounts of the individuals, while the *y*-axis shows the average detection rates.

For single-source DNA samples, we observed a decrease in the number of loci detected as DNA input decreased (Figure 3A). The detection of loci is expected to correlate with the quantity of DNA input rather than the number of contributors (Figure 4A). The detection rate of loci was as high as 93.95% in a 10 ng DNA mixture, but decreased to 81.18% when the DNA input was reduced to 0.05 ng. This decrease aligns with the results of the sensitivity study, further emphasizing the direct correlation between the detection rate and the quantity of DNA input. These findings underscore that the number of contributors does not affect the detection rate, and that a higher DNA input can result in a larger number of detected loci.

Higher input amounts have been shown to increase the detection rate, thereby providing more genetic markers for subsequent analysis. However, in mixture detection, the DNA amount of each contributor varies in proportion in each sample, leading to variations in typings even when the same DNA input is used. To minimize this difference, the contributor with the lowest DNA amount of was defined as the same person (individual B) in the unbalanced DNA mixtures. As expected, individual B consistently exhibited the lowest detection rate, except in Sample M37 (Figure 4B). Signals from low-contributing sources are prone to being masked by those from high contributors (van Oorschot et al., 2010). Similar to previous findings, more accurately typed loci were observed in the major contributors. Though researchers often describe detection rates in terms of component ratios, the significance of DNA input can be overlooked. Figure 4C demonstrates that while the ratio of components in mixtures remained constant, there was significant variation in detection rates. For example, individual B had a contribution proportion of 10% in 43 DNA mixtures, but the detection rate of the loci was inconsistent. These results suggest that, in addition to component proportions, another critical factor must influence contributor genotyping.

In the sensitivity analysis, the number of loci decreased with decreased DNA input (Figure 2B). The same trend in detection rate was observed in the mixture study. Within the range of 0.078 ng–0.156 ng DNA, approximately 99.68% of the contributor’s loci could be accurately typed, which was similar to the rate of 99.93% at 0.15625 ng DNA in the sensitivity study. For single source DNA samples, over 78.69% of the loci were correctly typed at an input of 0.00976563 ng. A comparable level (81.31%) was observed in the mixture study for contributors with DNA inputs between 0 and 0.001 ng (Figure 4C), indicating a similar detection limit between single- DNA and multi-sourced DNA samples. As detection success was closely linked to the DNA input of the minor contributor, we anticipated similar detection rates amongst the contributors in the balanced DNA mixtures. As expected, the contributors showed indistinguishable detection rates in the balanced 5- and 10-person DNA mixtures (Figure 4B).

### 3.5 Statistical parameters of the “FD multi-SNP mixture kit”

**FIGURE 5:**
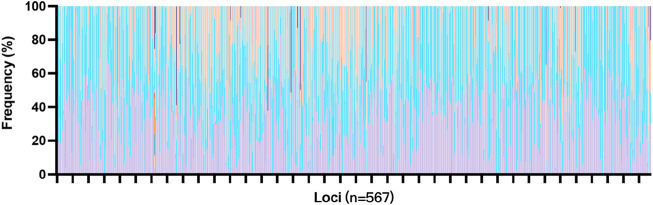
Allele frequencies of the 567 markers in the “FD multi-SNP Mixture Kit” within the Chinese Han population.

High-throughput SNP genotyping has been shown to be effective due to smaller amplicons, but the limited heterozygosity of SNP markers may make them less informative for certain purposes (Hares, 2015). Kidd et al. (Soundararajan et al., 2016) suggest that SNPs must contain multiple alleles to capture sufficient genetic diversity. This panel consists of 567 multi-SNPs, which is 30 times the size of the expanded Combined DNA Index System (CODIS) core loci, suggesting that the panel may have higher discrimination power. The 567 markers generated 1,855 unique allelotypes, and the corresponding allele frequencies are shown in Figure 1. Most of the loci had 2 to 4 unique allelotypes, with CHM020 being the most polymorphic locus with 16 unique genotypes, followed by CHM519 with 8 unique allelotypes. On the other hand, CHM462 and CHM085 showed poor polymorphism, with only one allelotype found among the 409 unrelated individuals. However, these loci accounted for only a small proportion of the panel (Additional file: Supplementary Table S1; Figure 5).

Other forensic statistical parameters, such as Hardy-Weinberg equilibrium (HWE), expected heterozygosity (He), observed heterozygosity (Ho), match probability (MP), discrimination power (DP), and power of exclusion (PE), were also determined. More than 98.06% (556/567) of the loci followed HWE, and 11 loci deviated from HWE after Bonferroni correction (*p* = 0.00008818). The average He and Ho were 0.55 ± 0.12 and 0.45 ± 0.12, respectively. The MP values ranged from 0.04 to 1, and DP values ranged from 0 to 0.96. The highest DP was found at CHM020 (0.96), and the highest PE was observed at CHM348 (1.00) (Additional file: Supplementary Table S2). Large panels are rich in genetic markers and are expected to provide more opportunities for genetic identification. However, large panels may also increase the likelihood of allelic dropouts (Shestak et al., 2021). In this study, the panel comprised 567 markers, with an average of 98.21% ± 1.44% of markers genotyped for each individual. Furthermore, 446 loci were stably detected in all 409 unrelated individuals, with combined power of exclusion (CPE), combined match probability (CMP), and total discrimination power (TDP) values of 1–0.00×10°, 0.00×10° and 1–0.00×10°, respectively, suggesting that this panel is powerful for the purpose of forensic identification.

## 4 Conclusion

In this study, our aim to develop novel genetic markers for the deconvolution of complex human DNA mixtures. We utilized the D value to evaluate the multi-SNP markers, which ultimately led to the creation of the “FD Multi-SNP Mixture Kit”. This kit features 567 multi-SNPs and is highly effective in DNA mixture detection. The high polymorphism and intra-locus balance across the loci may enhance the utility in mixture deconvolution. Our approach demonstrated exceptional sensitivity in analyzing both single-source and mixed DNA samples, underscoring its potential for identifying donors in low-yield samples.

## Data Availability

The datasets presented in this study can be found in NCBI Sequence Read Archive (SRA) repository, accession number PRJNA1163920 (https://www.ncbi.nlm.nih.gov/sra/PRJNA1163920).
